# Retraction for Hiramatsu et al., “The Mechanism of Pertussis Cough Revealed by the Mouse-Coughing Model”

**DOI:** 10.1128/mbio.03921-24

**Published:** 2025-02-05

**Authors:** Yukihiro Hiramatsu, Koichiro Suzuki, Takashi Nishida, Naoki Onoda, Takashi Satoh, Shizuo Akira, Masahito Ikawa, Hiroko Ikeda, Junzo Kamei, Sandra Derouiche, Makoto Tominaga, Yasuhiko Horiguchi

## RETRACTION

Volume 13, no. 2, e03197-21, 2022, https://doi.org/10.1128/mbio.03197-21. An investigation conducted by the corresponding author’s laboratory has concluded that the data shown in Fig. 3D–F, 4A–E and G, 5, S1, S2, S4, and S5 are the results of fabricated experiments or extensive falsification. An official investigation is ongoing at Osaka University, and certain results will require further verification by re-experimentation. Therefore, we are retracting the article and regret any inconvenience this may have caused.

